# Long-term Seizure Disorder Caused by a Dermoid Cyst with Catastrophic Developments

**DOI:** 10.7759/cureus.3272

**Published:** 2018-09-10

**Authors:** Yasaman Alam, Luke A Mugge, Jenna Purdy, Robert E Mrak, Jason Schroeder

**Affiliations:** 1 Neurosurgery, University of Toledo School of Medicine, Toledo, USA; 2 Surgery/Neurosurgery, University of Toledo Medical Center, Toledo, USA; 3 Pathology, University of Toledo Medical Center, Toledo, USA

**Keywords:** glioblastome multiforme, dermoid cyst, aneurysm, seizures, chronic inflammation, tumorigenesis

## Abstract

Glioblastoma multiforme (GBM) is a World Health Organization (WHO) grade IV primary malignant astrocytoma. Aneurysms are devastating intracranial neurovascular pathologies. Intracranial dermoid cysts are common, benign lesions which can be clinically silent or associated with seizure disorder.

We describe physically adjacent diagnoses of dermoid cyst, intracranial aneurysm, and GBM in a single patient. Records were collected and reviewed to compile the final clinical picture.

A 72-year-old male with a long history of seizure disorder, presented with new focal, unilateral neurological deficits. Radiographic evaluation including computed tomography (CT) and magnetic resonance imaging (MRI) demonstrated a dermoid cyst with an underlying developing GBM, which also, by happenstance, contained an aneurysm. During open surgical resection, multiple macroscopically distinct tissue types were noted. Histological analysis of tissue from each lesion confirmed the diagnoses including dermoid cyst, GBM, and aneurysm. Pathological analysis revealed the presence of extensive inflammatory cells throughout. Subsequent staining identified CD68 positive cells indicating a probable chronic inflammatory state.

Chronic inflammation resulting from the presence of a long term dermoid cyst and ongoing seizures may have led to dystrophic changes in adjacent vasculature and approximating glial tissues, inducing the formation of an aneurysm and a secondary GBM. Therefore, while benign in nature, dermoid cysts can be related to seizure disorder and may cause chronic inflammation in surrounding brain tissue.

## Introduction

Dermoid cysts are benign central nervous system (CNS) tumors derived from ectopic epithelial tissues [1]. These tumors are relatively uncommon, accounting for less than 1% of intracranial neoplasms [2,3]. Clinically, dermoid cysts can be benign, causing no significant symptomatology. Alternatively, when involving surrounding tissue, they can potentiate several disorders ranging from seizures and headaches to olfactory delusions [3]. Additionally, dermoid cysts can rupture and lead to both acute and chronic inflammation. The most common symptoms associated with rupture are seizures and headache [4]. Dermoid cysts can cause a number of pathological changes to the brain ranging from mass effect, excitotoxicity related to repetitive depolarization from seizures, and inflammation as a result of cystic content rupture. A well-adjusted inflammatory response does not lead to further deleterious effects for the adjacent tissues. However, chronic inflammation can predispose to oncogenic changes. The microenvironment of neoplasia has been shown to contain inflammatory cells which potentiate the chronic inflammatory state. Key players in this cancer-related inflammatory processes include T cells and tumor-associated macrophages with their associated cytokines including interleukin (IL)-1, IL-6, and tumor necrosis factor (TNF) [5,6].

Glioblastoma multiforme (GBM) is a grade IV astrocytoma according to World Health Organization (WHO) classification of primary malignant neoplasms of the central nervous system. The etiology of GBM development is not well understood, however, Urbanska et al. did reveal that incidence is slightly higher in Caucasian populations [7]. The total annual incidence of GBM is 4.13 cases per 100,000 worldwide with an average survival rate of 15 months post diagnosis [8,6]. While often seen clinically as an isolated pathology, several reports have identified the concomitant occurrence of GBM with intracranial aneurysm. From a clinical perspective, this association of GBM with aneurysms has significant implications as it could affect the treatment options and outcomes. Aneurysms can be located near the tumor or in remote locations, which change the surgical intervention from requiring two different sites of approach versus a single site. The occurrence of intracranial aneurysms in the general population is approximately 0.2–8.9%, and similarly Rushna et al. showed that the simultaneous occurrence of GBM, together with intracranial aneurysm, is 0.19–4% within the general population [8].

Here, we present a very rare case of GBM which was diagnosed via histopathological analysis with simultaneously adjacent dermoid cyst and aneurysm. Mechanistically, we hypothesize that chronic inflammation was the driver of tumorigenesis in this case.

## Case presentation

Past medical history and symptomatology

**Figure 1:**
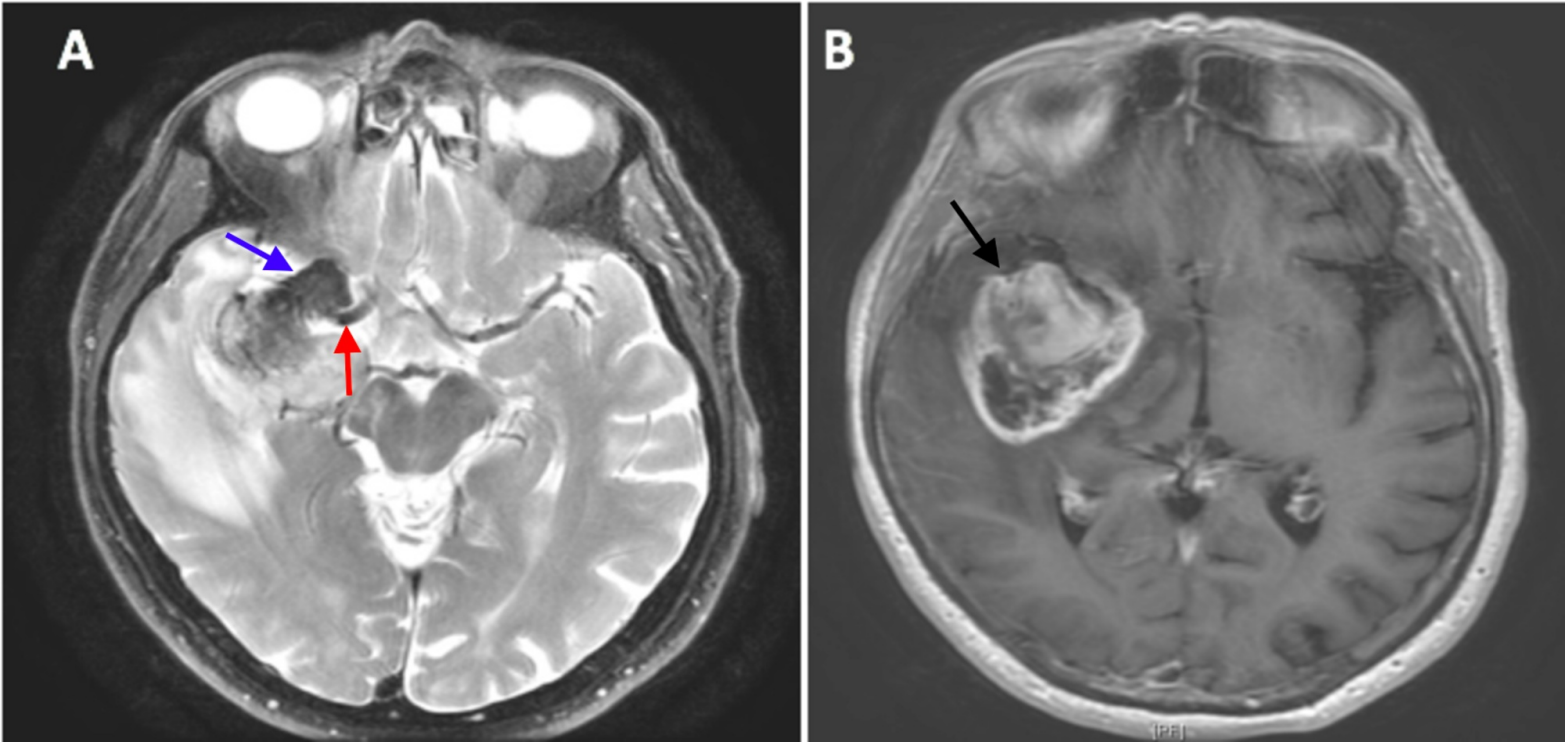
Magnetic resonance imaging (MRI) of patient's brain. (A) Axial T2 MRI with extramedullary lesion (blue arrow) adjacent to middle cerebral artery (red arrow); (B) T1 post-gad MRI with surround enhancing lesion (black arrow) with central necrosis consistent with Glioblastoma Multiforme.

A 72-year-old white male with a past medical history significant for hypertension, dyslipidemia, coronary artery disease status post-coronary artery bypass grafting, and chronic heavy smoking presented with a two decade history of seizure disorder due to an undefined brain lesion. At presentation, the patient had new deficits including slurred speech, ataxia, weakness, fatigue, and altered mental status. He had also lost weight over several months. He and his family denied any change in his seizures, new headaches, nausea, vomiting, or sensory changes. His initial head-CT showed a right temporal/frontal lobe mass with vasogenic edema, intralesional calcifications and 5 to 6 mm of midline shift with effacement of the temporal lobe sulci. Further investigation via magnetic resonance imaging (MRI) (Figures 1A-1B) revealed what appeared to be multiple different lesion types including both extramedullary and intramedullary masses. The extramedullary portion of the lesion was hypointense on T1 non-contrast MRI as well as T1 post-contrast MRI (Figure 1B) and appeared to be abutting the dura of the antero-medial middle temporal fossa. The intra-axial mass was hyperintense on T1 non-contrast imaging and showed diffusion restriction at its periphery with post-gadolinium enhancement of the immediately adjacent brain tissue. Finally, the proximal middle cerebral artery was coursing directly between these two areas of the lesion.

Surgical procedure

After a thorough discussion with the patient and family, the patient underwent right frontotemporal craniotomy with image guidance in order to obtain diagnosis and to resect the lesion. A pterional craniotomy large enough to expose the sylvian fissure and the adjacent frontal and temporal cortices was created and microscopic dissection was completed to open the sylvian fissure widely and to expose the insular cortex and the adjacent frontal and temporal areas. The extramedullary component of the mass was firm and partially calcified. This could not be mobilized away from the middle cerebral artery (MCA) trunk and had a clear border outside the brain tissue. The surrounding brain tissue was abnormally soft and showed macroscopic evidence of necrosis with traversing thrombosed vessels. The extramedullary lesion was opened sharply with microscissors and within this lesion, there appeared to be densely packed old hemorrhage and hemosiderin deposition. A portion of the central contents of the lesion was sampled and sent for pathology as well as a piece of the lesion wall. The findings of old clotted blood within this lesion and dense adherence to the MCA vessels raised clinical concerns for a partially thrombosed aneurysm and it was decided to allow the remainder of the lesion to remain intact. Subsequently, surrounding brain tissue was removed and intra-operative pathological analysis from this tissue resulted in the diagnosis of a high-grade glioma. The suspected GBM was then debulked to the edge of its enhancing border.

Histopathological findings

**Figure 2:**
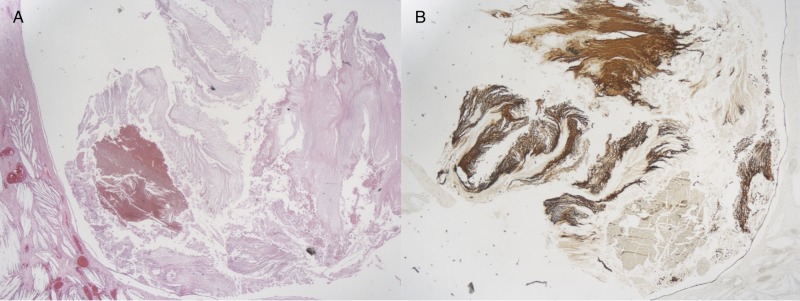
Post resection pathology of dermoid cyst. (A) Epidermal cysts with thin lining of squamous cells that is filled with squamous debris (H&E, X40); (B) squamous cell and debris are pancytokeratin positive (X40).

The first biopsy of the right insular tumor (Figures 2A-2B) showed structures consistent with a dermoid cyst. This biopsy included old blood/hemorrhage with keratinaceous debris. There was no evidence of organization and no epithelium was identified. Figure 2A depicts a mass with thin lining of squamous cells that is filled with squamous debris indicating the presence of dermoid cyst. Squamous cell and debris are pancytokeratin positive (Figure 2B).

**Figure 3:**
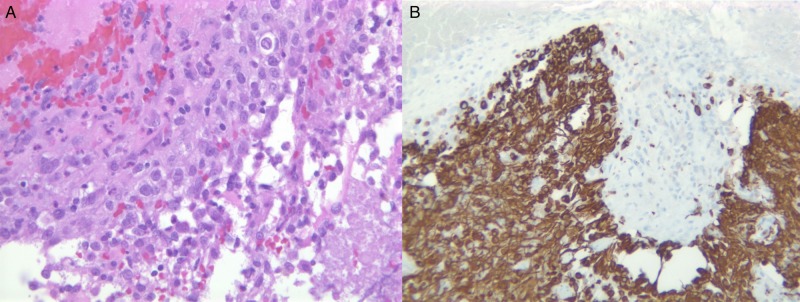
Post-resection pathology of Glioblastoma multiforme. (A) Irregular malignant cells, with some necrotic tissues; (B) Malignant cells positive for Glial fibrillary acidic protein (GFAP).

The second biopsy (Figures 3A-3B) area taken from the right insular tumor demonstrated features consistent with a GBM including increased cellularity, nuclear pleomorphism, necrosis and vascular proliferation. It shows highly cellular and irregular malignant cells which are positive for Glial fibrillary acidic protein (GFAP) (Figure 3B), with necrosis indicating the presence of GBM.

**Figure 4:**
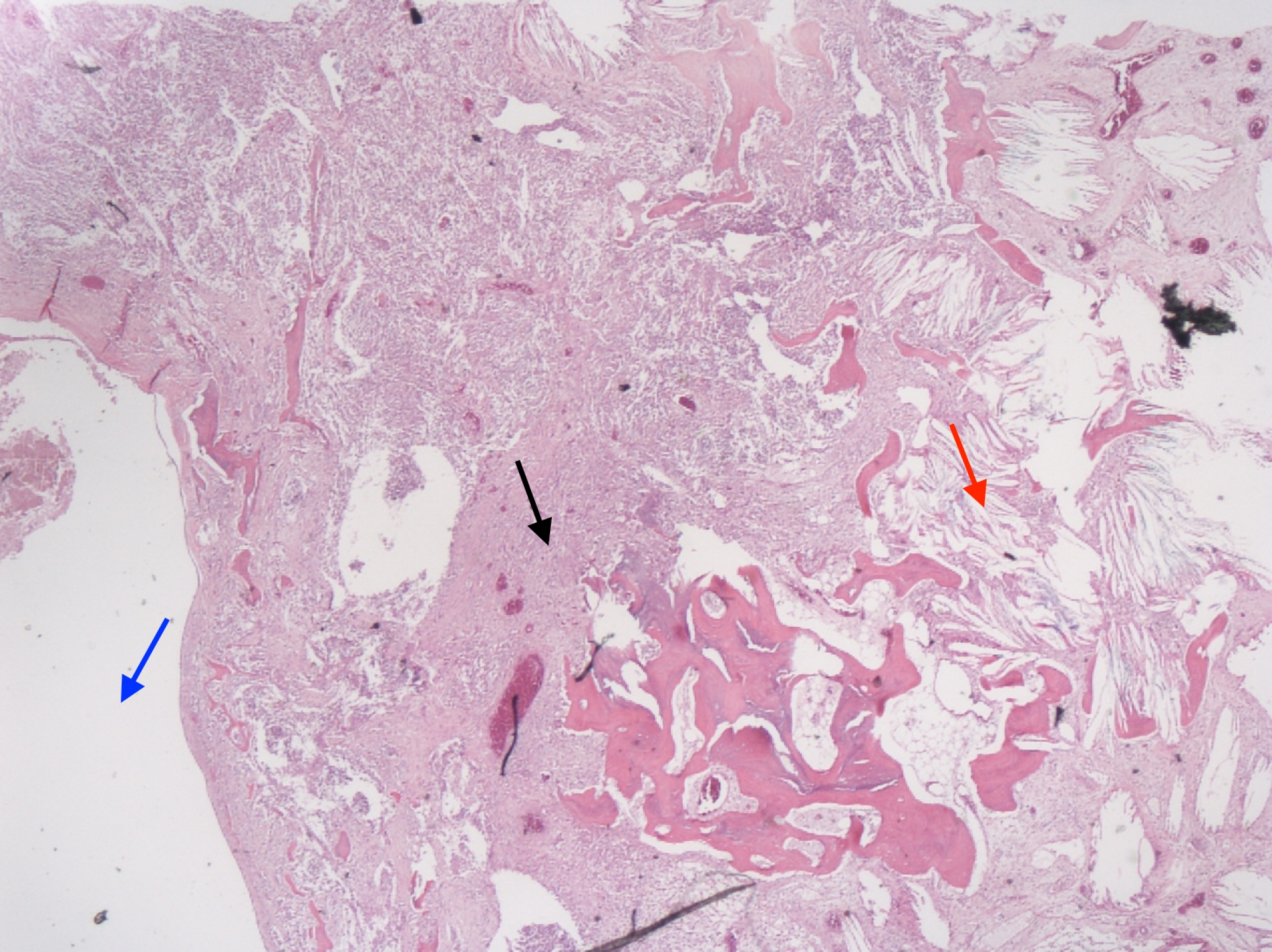
Pathological analysis of all three tissues post resection. (Blue Arrow) Dermoid cyst; (Black Arrow) Glioblastoma multiforme hypercellular area with malignant appearing cells; (Red Arrow) Wall of the aneurysm with cholesterol clefts (X40).

Finally, the third biopsy (Figure 4) from the extramedullary portion of the lesion adjacent to the middle cerebral artery was determined to be consistent with an aneurysm with features including dense collagenous tissue with calcification and remodeling into bony trabeculae. In this figure, we can see all three structures next to one another.

**Figure 5:**
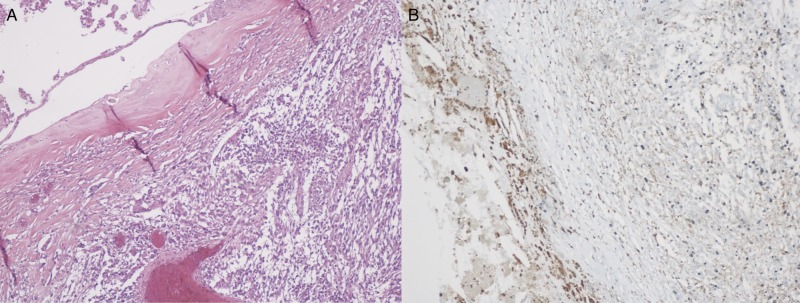
Testing the post-resection tissue for inflammatory cell, macrophages. (A) Cyst wall upper left with adjacent Glioblastoma multiforme tumor lower right. (H&E, X100); (B) CD 68 highlighting macrophages within the cyst wall and the tumor (X100).

**Figure 6:**
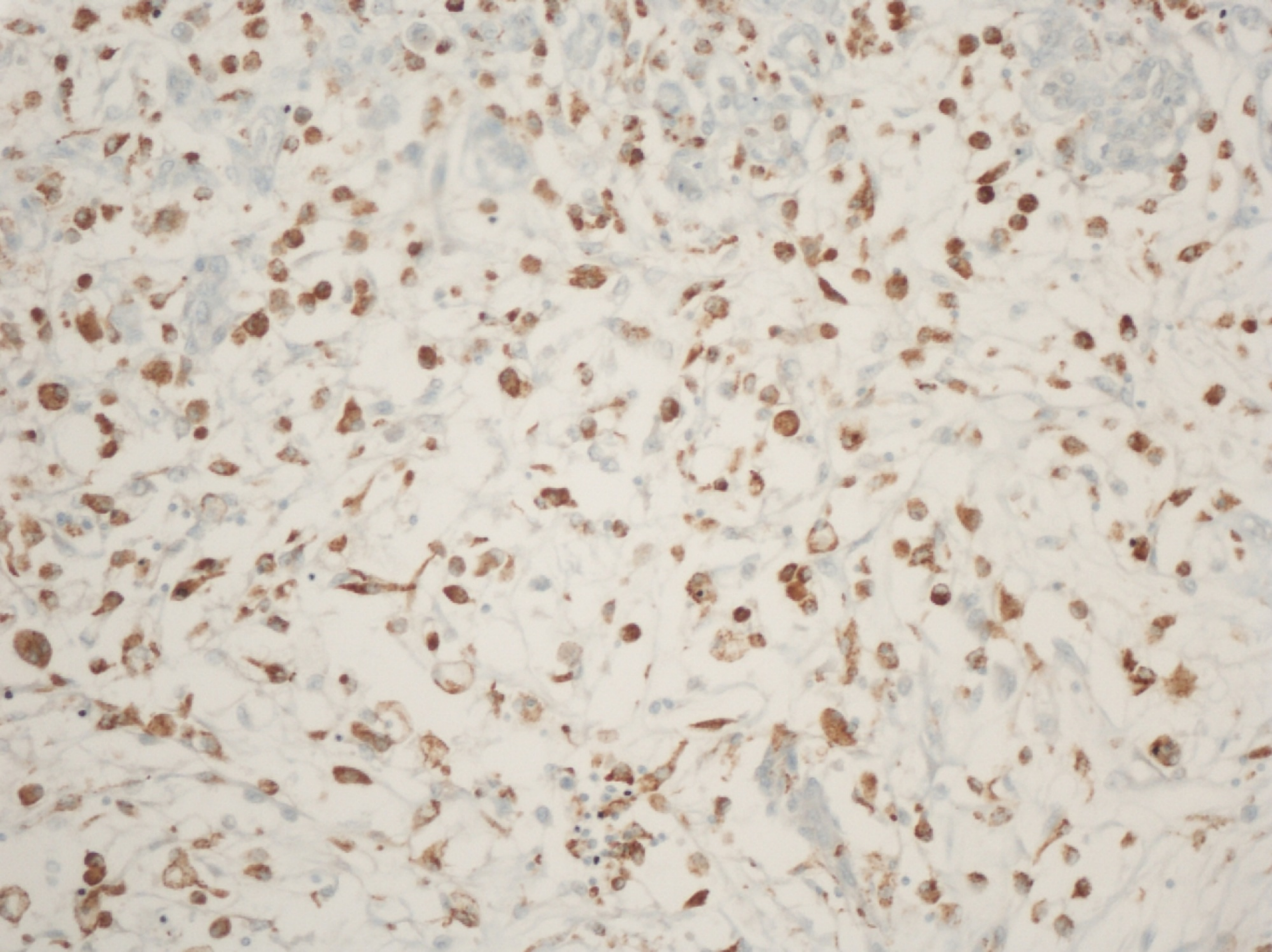
Testing the post-resection glioblastoma tissue for inflammatory cell, macrophages. CD68 positive Glioblastoma multiforme (X200).

In order to examine the presence of inflammatory cells within the lesion, specific stains were applied to the aforementioned specimens. Figure 5 and Figure 6 depict a slide stained for CD 68 to examine for macrophages. As shown, the stain was positive for CD 68, highlighting macrophages within the cyst wall and the tumor, indicating the presence of inflammatory cells.

## Discussion

For this patient, the biopsied tissue confirms the presence of multiple co-existing lesions – a benign tumor (dermoid cyst), an aneurysm, and a highly malignant neoplasm (GBM). In several case reports, GBMs have previously been reported to be present along with meningioma, but no reports were found identifying GBM co-existing with a dermoid cyst. In one case report by Sahuc et al., a patient had a meningioma treated with complete resection who suffered two subsequent recurrences, which ultimately were histopathologically confirmed to be GBM [9]. Suzuki et al. reported a similar case of simultaneous formation of GBM with meningioma. In that report, preoperative MRI indicated a single lesion, but postoperative histology identified that there was GBM adjacent to meningioma [10].

Dermoid cysts have been reported to be associated with new onset of seizure disorder. Potential sequelae of dermoid cysts include rupture leading to inflammation or growth with expansion leading to compression of neural tissue, both of which can cause seizures [1,4].

Inflammation is a known and common component of tissue repair, malignant progression, and infection resolution. Within the CNS, tissue repair begins with microglia activation which results from released inflammatory mediator triggers as part of the response to axonal injury or traumatic change to the local environment. Once activated, microglia promote T-cell infiltration through inflammatory cytokine production, culminating in the development of an inflammatory response [11-13]. In addition, cytokines lead to maturation of macrophages into one of two distinct phenotypes, M1 and M2. M1 macrophages have pro-inflammatory activities and release IL-12 and TNF alpha while M2 macrophages have tumorigenesis activities through secretion of IL10 and tumor growth factor (TGF) beta. If this acute inflammatory microenvironment fails to resolve, chronic inflammation will lead to progression of existing CNS tumors and the development of a new CNS neoplasms [14].

In this patient, long term seizure disorder was likely due to chronic inflammation induced by a yet unidentified brain lesion which was subsequently determined to be a dermoid cyst. Histological analysis revealed the presence of CD68 positive cells confirming unresolved inflammation. Given the history and ultimate histological identification of multiple adjacent lesions, chronic inflammation due to dermoid cyst/seizures could potentially have contributed to induction of the GBM.

This case highlights how seemingly benign lesions, such as dermoid cysts, can potentially lead to chronic inflammation, and thus possibly contribute to the development of significantly worse CNS pathologies. This report supports the need for a detailed assessment, potential aggressive treatment, and diligent follow-up even for patients with pathologies that are considered benign (i.e., dermoid cysts) in order to help avoid other more severe outcomes.

## Conclusions

Intracranial neoplasms are commonly encountered within the field of neurosurgery. Understanding the implications for different types of tumors is central to the provision of effective and appropriate care. Having appreciation for the theoretical malignant potential of seemingly benign lesions is central to the overall clinical decision-making process. With this in mind, there is a role for aggressive treatment and diligent follow-up when dermoid cysts are encountered in order to avoid potentially worsened outcomes. This case further highlights the need to remain open-minded concerning the ultimate diagnosis regardless of what seems clear pathologically or radiologically at presentation in addition to revealing that long term inflammation may alter local micro-environments, potentiating neoplastic development.
